# Bis{[6-meth­oxy-2-(4-methyl­phen­yl)iminiometh­yl]phenolate-κ^2^ *O*,*O*′}tris­(nitrato-κ^2^ *O*,*O*′)europium(III)

**DOI:** 10.1107/S1600536810042194

**Published:** 2010-10-23

**Authors:** Hang-Ming Guo

**Affiliations:** a Jinhua College of Vocation and Technology, Jinhua, Zhejiang 321017, People’s Republic of China

## Abstract

The crystal structure of title compound, [Eu(NO_3_)_3_(C_15_H_15_NO_2_)_2_], contains two Schiff base 6-meth­oxy-2-[(4-methyl­phen­yl)imino­meth­yl]phenolate (*L*) ligands and three independent nitrate ions that chelate to the europium(III) ion *via* the O atoms. The coordination number of the Eu^III^ ion is ten. The *L* ligands chelate with a strong Eu—O(deprotonated phenolate) bond and a weak Eu—O(meth­oxy) contact, the latter can be inter­preted as the apices of the bicapped square-anti­prismatic Eu^III^ polyhedron. Intra­molecular N—H⋯O hydrogen bonds occur.

## Related literature

For Schiff base ligands derived from *o*-vanillin and aniline and their rare earth complexes, see: Burrows & Bailar (1966 ▶); Li *et al.* (2008 ▶); Liu *et al.* (2009 ▶); Xian *et al.* (2008 ▶); Zhao *et al.* (2005 ▶, 2007 ▶).
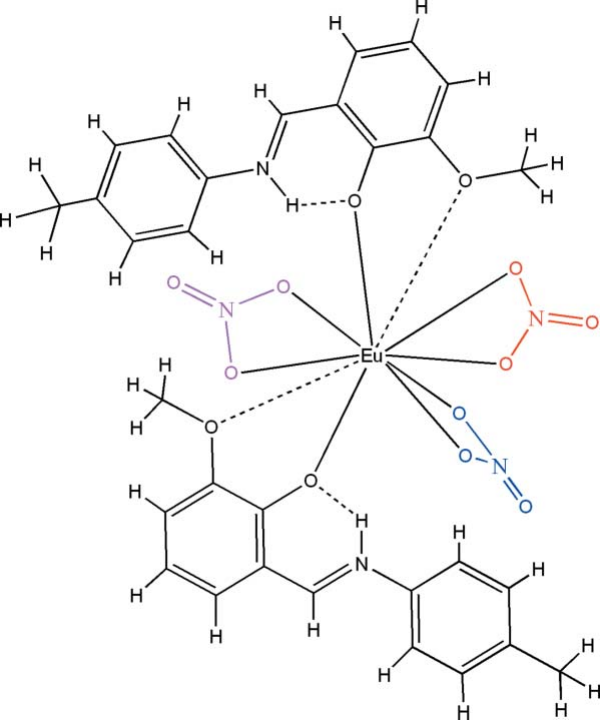


## Experimental

### 

#### Crystal data

[Eu(NO_3_)_3_(C_15_H_15_NO_2_)_2_]
*M* *_r_* = 820.55
Triclinic, 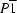

*a* = 9.7603 (7) Å
*b* = 10.0250 (7) Å
*c* = 18.4227 (16) Å
α = 98.165 (6)°
β = 101.665 (6)°
γ = 106.681 (4)°
*V* = 1652.2 (2) Å^3^
*Z* = 2
Mo *K*α radiation
μ = 1.97 mm^−1^
*T* = 296 K
0.18 × 0.09 × 0.06 mm

#### Data collection

Bruker APEXII area-detector diffractometer
Absorption correction: multi-scan (*SADABS*; Sheldrick, 1996 ▶) *T*_min_ = 0.801, *T*_max_ = 0.892
26273 measured reflections
7610 independent reflections
5570 reflections with *I* > 2σ(*I*)
*R*_int_ = 0.051

#### Refinement

*R*[*F* ^2^ > 2σ(*F* ^2^)] = 0.040
*wR*(*F* ^2^) = 0.094
*S* = 0.99
7610 reflections
442 parameters
H-atom parameters constrained
Δρ_max_ = 0.85 e Å^−3^
Δρ_min_ = −0.69 e Å^−3^

### 

Data collection: *APEX2* (Bruker, 2006 ▶); cell refinement: *SAINT* (Bruker, 2006 ▶); data reduction: *SAINT*; program(s) used to solve structure: *SHELXS97* (Sheldrick, 2008 ▶); program(s) used to refine structure: *SHELXL97* (Sheldrick, 2008 ▶); molecular graphics: *SHELXTL* (Sheldrick, 2008 ▶); software used to prepare material for publication: *SHELXL97*.

## Supplementary Material

Crystal structure: contains datablocks I, global. DOI: 10.1107/S1600536810042194/hg2728sup1.cif

Structure factors: contains datablocks I. DOI: 10.1107/S1600536810042194/hg2728Isup2.hkl

Additional supplementary materials:  crystallographic information; 3D view; checkCIF report

## Figures and Tables

**Table 1:** Hydrogen-bond geometry (Å, °)

*D*—H⋯*A*	*D*—H	H⋯*A*	*D*⋯*A*	*D*—H⋯*A*
N1—H1*A*⋯O1	0.86	1.95	2.634 (4)	136
N2—H2*A*⋯O3	0.86	1.86	2.569 (4)	139

**Fig. 1.**
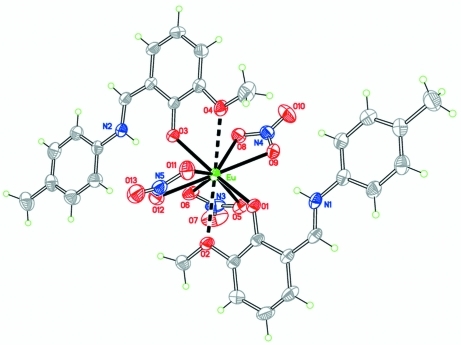
The molecular structure of the title complex, showing the atom-labeling scheme. Displacement ellipsoids are drawn at the 30% probability level.

**Fig. 2.**
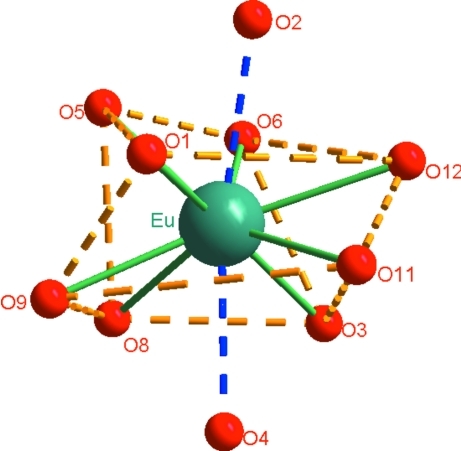
The coordination environment of the Europium(III) atom, showing the bicapped square antiprism.

**Fig. 3.**
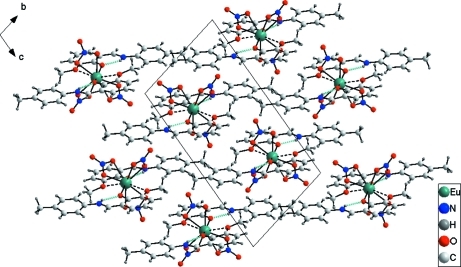
The stacking plot of the title compound, showing H-bond interactions (dashed lines) and π···π stacking interactions.

## supplementary crystallographic information

### Comment

It has well been confirmed that Schiff bases are important in multiple fields such as chemistry and biochemistry owing to their biological activities (Zhao *et al.*, 2005). Schiff base complexes prepared by ligands from substituted *o*-vanillin have been absorbed considerable attention in the past decades due to the intriguing biological activities of *o*-vanillin and the convenience in Schiff bases synthesis (Burrows & Bailar, 1966). Interested in this field, we have been engaged in a major effort directed toward the development of syntheses of new analogous Schiff bases derived from *o*-vanillin and their rare metal complexes. In a few of articles we have reported our partial research results (Zhao *et al.*, 2007; Xian *et al.* 2008; Li *et al.* 2008; Liu *et al.* 2009). Herein, we describe a new Eu^III^ complex.

The structure of the title complex is shown in Fig.1, and the coordination environment of Eu^III^ is shown in Fig. 2. In this complex the Eu^III^ is eight-coordinated by O atoms, six of which come from three nitrate ions and two come from the Schiff base ligands (H*L*). The H*L* ligands coordinate to the Eu^III^ ion using oxygen atoms from deprotonated phenolic hydroxyl groups. The ten Eu—O bond distances are listed in Table 1 (including weak Eu—O interactions). The distances between Eu^III^ and methoxyl O atoms (2.743Å and 2.748Å for Eu—O2 and Eu—O4) are similar with reported complexe (Zhao *et al.*, 2007).

The hydrogen bonds and π···π weak non-covalent interactions lend stability to the structure. The hydrogen bonds are listed in Table 2 and the stacking plot of this compound is shown in Fig. 3. There is π···π interactions exist in the crystal between symmetry-related molecules. In H*L* ligands, the proton of the phenolic hydroxyl group is considered to have transferred to *N*-imine atom, which involving in an intramolecular hydrogen bond (Table 2).

### Experimental

Reagents and solvents used were of commercially available quality and without purified before using. The Schiff base ligand 2-[(4- methylphenyl)iminomethyl]-6-methoxy-phenol was prepared by condensation of *o*-vanillin and *p*-methylaniline with a high yield and which was purified by recrystallization in ethanol. The compound (1) was obtained by adding Eu(NO_3_)_3_ (1 mmol, dissolved in ethanol) to *N*-salicylidene-*p*-toluidine (2 mmol) in ethanol solution. The mixture solution was stirred at room temperature for 8 h to obtain a purplish red solution. At last, the deposit was filtered out and the solution was kept for evaporating. The red crystal was formed after several days.

### Refinement

The structure was solved by direct methods and successive Fourier difference synthesis. The H atoms bonded to C and N atoms were positioned geometrically and refined using a riding model [aliphatic C—H =0.96 Å (*U*_iso_(H) = 1.5*U*_eq_(C)), aromatic C—H = 0.93 Å (*U*_iso_(H) = 1.2*U*_eq_(C)) and N—H = 0.86 Å with *U*_iso_(H) = 1.2*U*_eq_(N).

### Crystal data

**Table:**

[Eu(NO_3_)_3_(C_15_H_15_NO_2_)_2_]	*Z* = 2
*M**_r_* = 820.55	*F*(000) = 824
Triclinic, *P*1	*D*_x_ = 1.649 Mg m^−^^3^
Hall symbol: -P 1	Mo *K*α radiation, λ = 0.71073 Å
*a* = 9.7603 (7) Å	Cell parameters from 4647 reflections
*b* = 10.0250 (7) Å	θ = 1.2–27.7°
*c* = 18.4227 (16) Å	µ = 1.97 mm^−^^1^
α = 98.165 (6)°	*T* = 296 K
β = 101.665 (6)°	Block, red
γ = 106.681 (4)°	0.18 × 0.09 × 0.06 mm
*V* = 1652.2 (2) Å^3^	

### Data collection

**Table:**

Bruker APEXII area-detector diffractometer	7610 independent reflections
Radiation source: fine-focus sealed tube	5570 reflections with *I* > 2σ(*I*)
graphite	*R*_int_ = 0.051
phi and ω scans	θ_max_ = 27.7°, θ_min_ = 1.2°
Absorption correction: multi-scan (*SADABS*; Sheldrick, 1996)	*h* = −12→11
*T*_min_ = 0.801, *T*_max_ = 0.892	*k* = −12→13
26273 measured reflections	*l* = −23→24

### Refinement

**Table:**

Refinement on *F*^2^	Primary atom site location: structure-invariant direct methods
Least-squares matrix: full	Secondary atom site location: difference Fourier map
*R*[*F*^2^ > 2σ(*F*^2^)] = 0.040	Hydrogen site location: inferred from neighbouring sites
*wR*(*F*^2^) = 0.094	H-atom parameters constrained
*S* = 0.99	*w* = 1/[σ^2^(*F*_o_^2^) + (0.046*P*)^2^] where *P* = (*F*_o_^2^ + 2*F*_c_^2^)/3
7610 reflections	(Δ/σ)_max_ = 0.001
442 parameters	Δρ_max_ = 0.85 e Å^−^^3^
0 restraints	Δρ_min_ = −0.69 e Å^−^^3^

### Special details

**Table:**

Geometry. All e.s.d.'s (except the e.s.d. in the dihedral angle between two l.s. planes) are estimated using the full covariance matrix. The cell e.s.d.'s are taken into account individually in the estimation of e.s.d.'s in distances, angles and torsion angles; correlations between e.s.d.'s in cell parameters are only used when they are defined by crystal symmetry. An approximate (isotropic) treatment of cell e.s.d.'s is used for estimating e.s.d.'s involving l.s. planes.
Refinement. Refinement of *F*^2^ against ALL reflections. The weighted *R*-factor *wR* and goodness of fit *S* are based on *F*^2^, conventional *R*-factors *R* are based on *F*, with *F* set to zero for negative *F*^2^. The threshold expression of *F*^2^ > σ(*F*^2^) is used only for calculating *R*-factors(gt) *etc*. and is not relevant to the choice of reflections for refinement. *R*-factors based on *F*^2^ are statistically about twice as large as those based on *F*, and *R*- factors based on ALL data will be even larger.

### Fractional atomic coordinates and isotropic or equivalent isotropic displacement parameters (Å^2^)

**Table:**

	*x*	*y*	*z*	*U*_iso_*/*U*_eq_	
Eu	0.96447 (2)	0.31215 (2)	0.252484 (12)	0.04432 (9)	
N1	0.5030 (4)	−0.0343 (4)	0.2208 (2)	0.0516 (9)	
H1A	0.5916	0.0121	0.2203	0.062*	
N2	1.4594 (4)	0.5536 (4)	0.27769 (19)	0.0507 (9)	
H2A	1.3707	0.5195	0.2821	0.061*	
N3	0.9483 (6)	0.5918 (5)	0.2364 (3)	0.0750 (13)	
N4	0.8421 (5)	0.2288 (4)	0.0886 (2)	0.0586 (10)	
N5	1.1222 (4)	0.2644 (5)	0.3940 (2)	0.0631 (10)	
O1	0.7425 (3)	0.1902 (3)	0.27686 (16)	0.0535 (7)	
O2	0.8756 (3)	0.4409 (3)	0.36760 (17)	0.0578 (8)	
O3	1.2074 (3)	0.3557 (3)	0.24344 (16)	0.0523 (7)	
O4	1.0359 (3)	0.0995 (3)	0.17308 (18)	0.0579 (8)	
O5	0.8301 (4)	0.4845 (4)	0.2152 (2)	0.0757 (10)	
O6	1.0654 (4)	0.5683 (3)	0.2656 (2)	0.0643 (9)	
O7	0.9460 (6)	0.7099 (5)	0.2303 (3)	0.1282 (19)	
O8	0.9612 (4)	0.3321 (3)	0.11866 (17)	0.0602 (8)	
O9	0.7730 (3)	0.1730 (3)	0.13420 (18)	0.0615 (8)	
O10	0.7985 (5)	0.1845 (4)	0.0214 (2)	0.0947 (13)	
O11	1.0396 (4)	0.1640 (4)	0.3384 (2)	0.0691 (9)	
O12	1.1413 (4)	0.3890 (4)	0.38578 (18)	0.0634 (8)	
O13	1.1807 (4)	0.2359 (4)	0.4530 (2)	0.0827 (11)	
C1	0.5234 (4)	0.1554 (5)	0.3210 (2)	0.0496 (10)	
C2	0.6699 (4)	0.2369 (4)	0.3215 (2)	0.0448 (10)	
C3	0.7328 (5)	0.3710 (5)	0.3714 (2)	0.0483 (10)	
C4	0.6600 (6)	0.4224 (5)	0.4186 (3)	0.0622 (12)	
H4A	0.7053	0.5112	0.4513	0.075*	
C5	0.5163 (6)	0.3401 (6)	0.4177 (3)	0.0732 (15)	
H5A	0.4657	0.3751	0.4493	0.088*	
C6	0.4511 (5)	0.2102 (6)	0.3708 (3)	0.0690 (14)	
H6A	0.3566	0.1560	0.3714	0.083*	
C7	0.4493 (5)	0.0238 (5)	0.2709 (3)	0.0531 (11)	
H7A	0.3547	−0.0257	0.2736	0.064*	
C8	0.9493 (6)	0.5808 (5)	0.4148 (3)	0.0651 (13)	
H8A	0.8936	0.5988	0.4502	0.098*	
H8B	0.9572	0.6505	0.3838	0.098*	
H8C	1.0465	0.5867	0.4420	0.098*	
C9	0.4335 (5)	−0.1648 (5)	0.1669 (2)	0.0511 (11)	
C10	0.3006 (5)	−0.2604 (5)	0.1683 (3)	0.0601 (12)	
H10A	0.2545	−0.2406	0.2061	0.072*	
C11	0.2364 (5)	−0.3845 (5)	0.1140 (3)	0.0642 (13)	
H11A	0.1460	−0.4467	0.1153	0.077*	
C12	0.3016 (6)	−0.4197 (5)	0.0579 (3)	0.0631 (12)	
C13	0.4352 (6)	−0.3230 (6)	0.0580 (3)	0.0758 (15)	
H13A	0.4820	−0.3436	0.0207	0.091*	
C14	0.5010 (5)	−0.1980 (5)	0.1110 (3)	0.0680 (14)	
H14A	0.5910	−0.1355	0.1095	0.082*	
C15	0.2269 (7)	−0.5540 (6)	−0.0017 (3)	0.0884 (18)	
H15A	0.1359	−0.6061	0.0083	0.133*	
H15B	0.2906	−0.6114	−0.0009	0.133*	
H15C	0.2062	−0.5307	−0.0506	0.133*	
C16	1.4000 (4)	0.3414 (4)	0.1847 (2)	0.0459 (10)	
C17	1.2577 (4)	0.2845 (4)	0.1960 (2)	0.0429 (9)	
C18	1.1705 (5)	0.1454 (4)	0.1551 (2)	0.0486 (10)	
C19	1.2195 (5)	0.0708 (5)	0.1046 (3)	0.0569 (11)	
H19A	1.1592	−0.0197	0.0776	0.068*	
C20	1.3599 (5)	0.1289 (5)	0.0929 (3)	0.0610 (12)	
H20A	1.3925	0.0771	0.0580	0.073*	
C21	1.4479 (5)	0.2602 (5)	0.1323 (3)	0.0561 (11)	
H21A	1.5416	0.2977	0.1248	0.067*	
C22	1.4960 (5)	0.4756 (5)	0.2272 (2)	0.0506 (10)	
H22A	1.5895	0.5100	0.2189	0.061*	
C23	0.9464 (6)	−0.0471 (5)	0.1409 (4)	0.0907 (19)	
H23A	1.0022	−0.0954	0.1165	0.136*	
H23B	0.8594	−0.0506	0.1043	0.136*	
H23C	0.9179	−0.0929	0.1804	0.136*	
C24	1.5452 (5)	0.6864 (4)	0.3263 (2)	0.0493 (10)	
C25	1.6818 (5)	0.7649 (5)	0.3177 (3)	0.0595 (12)	
H25A	1.7187	0.7318	0.2786	0.071*	
C26	1.7611 (6)	0.8923 (5)	0.3679 (3)	0.0680 (14)	
H26A	1.8524	0.9449	0.3624	0.082*	
C27	1.7085 (6)	0.9442 (5)	0.4266 (3)	0.0659 (13)	
C28	1.5720 (6)	0.8642 (5)	0.4324 (3)	0.0755 (15)	
H28A	1.5337	0.8975	0.4709	0.091*	
C29	1.4908 (5)	0.7368 (5)	0.3831 (3)	0.0654 (13)	
H29A	1.3990	0.6850	0.3884	0.078*	
C30	1.7989 (7)	1.0827 (5)	0.4829 (3)	0.0893 (18)	
H30A	1.7454	1.1012	0.5194	0.134*	
H30B	1.8170	1.1593	0.4564	0.134*	
H30C	1.8915	1.0752	0.5084	0.134*	

### Atomic displacement parameters (Å^2^)

**Table:**

	*U*^11^	*U*^22^	*U*^33^	*U*^12^	*U*^13^	*U*^23^
Eu	0.03130 (12)	0.04488 (13)	0.05244 (14)	0.00468 (9)	0.01419 (9)	0.00783 (9)
N1	0.0319 (18)	0.057 (2)	0.061 (2)	0.0053 (17)	0.0129 (17)	0.0180 (18)
N2	0.0349 (19)	0.054 (2)	0.057 (2)	0.0092 (17)	0.0097 (16)	0.0059 (17)
N3	0.100 (4)	0.069 (3)	0.087 (3)	0.041 (3)	0.059 (3)	0.034 (3)
N4	0.063 (3)	0.060 (2)	0.053 (3)	0.027 (2)	0.009 (2)	0.007 (2)
N5	0.052 (2)	0.089 (3)	0.057 (3)	0.028 (2)	0.022 (2)	0.021 (2)
O1	0.0402 (16)	0.0565 (18)	0.0618 (18)	0.0086 (14)	0.0225 (14)	0.0078 (14)
O2	0.0434 (17)	0.0566 (18)	0.066 (2)	0.0057 (15)	0.0238 (15)	−0.0005 (15)
O3	0.0370 (16)	0.0557 (17)	0.0576 (18)	0.0094 (14)	0.0150 (13)	−0.0007 (14)
O4	0.0417 (17)	0.0518 (18)	0.072 (2)	0.0033 (14)	0.0157 (15)	0.0111 (15)
O5	0.065 (2)	0.096 (3)	0.087 (3)	0.044 (2)	0.032 (2)	0.034 (2)
O6	0.058 (2)	0.0508 (19)	0.091 (2)	0.0152 (16)	0.0378 (19)	0.0172 (17)
O7	0.181 (5)	0.090 (3)	0.186 (5)	0.085 (3)	0.108 (4)	0.083 (3)
O8	0.059 (2)	0.0570 (19)	0.0600 (19)	0.0094 (17)	0.0190 (16)	0.0112 (15)
O9	0.0416 (17)	0.068 (2)	0.063 (2)	0.0055 (15)	0.0131 (15)	0.0037 (16)
O10	0.120 (3)	0.095 (3)	0.052 (2)	0.032 (3)	0.002 (2)	−0.003 (2)
O11	0.062 (2)	0.063 (2)	0.072 (2)	0.0077 (18)	0.0094 (18)	0.0177 (18)
O12	0.068 (2)	0.065 (2)	0.0555 (19)	0.0254 (18)	0.0107 (16)	0.0045 (16)
O13	0.078 (3)	0.123 (3)	0.063 (2)	0.047 (2)	0.0180 (19)	0.037 (2)
C1	0.036 (2)	0.058 (3)	0.060 (3)	0.015 (2)	0.019 (2)	0.020 (2)
C2	0.040 (2)	0.056 (3)	0.047 (2)	0.018 (2)	0.0197 (19)	0.022 (2)
C3	0.041 (2)	0.053 (3)	0.053 (3)	0.014 (2)	0.016 (2)	0.016 (2)
C4	0.064 (3)	0.067 (3)	0.058 (3)	0.024 (3)	0.021 (2)	0.008 (2)
C5	0.065 (3)	0.080 (4)	0.086 (4)	0.027 (3)	0.043 (3)	0.011 (3)
C6	0.048 (3)	0.079 (4)	0.089 (4)	0.018 (3)	0.037 (3)	0.024 (3)
C7	0.037 (2)	0.060 (3)	0.067 (3)	0.011 (2)	0.023 (2)	0.023 (2)
C8	0.062 (3)	0.051 (3)	0.070 (3)	0.008 (2)	0.016 (2)	−0.003 (2)
C9	0.043 (2)	0.051 (3)	0.059 (3)	0.014 (2)	0.009 (2)	0.019 (2)
C10	0.046 (3)	0.064 (3)	0.066 (3)	0.003 (2)	0.022 (2)	0.019 (2)
C11	0.055 (3)	0.060 (3)	0.069 (3)	0.003 (2)	0.014 (3)	0.024 (3)
C12	0.055 (3)	0.061 (3)	0.064 (3)	0.012 (2)	0.006 (2)	0.011 (2)
C13	0.060 (3)	0.089 (4)	0.077 (4)	0.025 (3)	0.022 (3)	0.004 (3)
C14	0.047 (3)	0.071 (3)	0.076 (3)	0.006 (2)	0.020 (3)	0.004 (3)
C15	0.085 (4)	0.079 (4)	0.081 (4)	0.018 (3)	0.004 (3)	−0.002 (3)
C16	0.037 (2)	0.050 (2)	0.053 (2)	0.0172 (19)	0.0125 (19)	0.012 (2)
C17	0.036 (2)	0.048 (2)	0.045 (2)	0.0147 (19)	0.0102 (18)	0.0102 (18)
C18	0.043 (2)	0.049 (2)	0.053 (3)	0.013 (2)	0.012 (2)	0.013 (2)
C19	0.057 (3)	0.052 (3)	0.060 (3)	0.021 (2)	0.010 (2)	0.007 (2)
C20	0.063 (3)	0.069 (3)	0.061 (3)	0.033 (3)	0.026 (2)	0.006 (2)
C21	0.045 (3)	0.069 (3)	0.063 (3)	0.024 (2)	0.024 (2)	0.017 (2)
C22	0.038 (2)	0.061 (3)	0.056 (3)	0.018 (2)	0.014 (2)	0.015 (2)
C23	0.064 (4)	0.052 (3)	0.150 (6)	0.006 (3)	0.026 (4)	0.029 (3)
C24	0.041 (2)	0.045 (2)	0.054 (3)	0.009 (2)	0.005 (2)	0.009 (2)
C25	0.045 (3)	0.059 (3)	0.067 (3)	0.006 (2)	0.016 (2)	0.011 (2)
C26	0.050 (3)	0.056 (3)	0.088 (4)	0.004 (2)	0.011 (3)	0.024 (3)
C27	0.064 (3)	0.047 (3)	0.071 (3)	0.009 (2)	−0.001 (3)	0.011 (2)
C28	0.076 (4)	0.062 (3)	0.077 (4)	0.010 (3)	0.024 (3)	0.001 (3)
C29	0.053 (3)	0.055 (3)	0.080 (3)	0.005 (2)	0.023 (3)	0.004 (2)
C30	0.086 (4)	0.062 (3)	0.089 (4)	0.004 (3)	−0.004 (3)	−0.002 (3)

### Geometric parameters (Å, °)

**Table:**

Eu—O1	2.328 (3)	C8—H8B	0.9600
Eu—O3	2.329 (3)	C8—H8C	0.9600
Eu—O6	2.429 (3)	C9—C14	1.381 (6)
Eu—O11	2.464 (3)	C9—C10	1.381 (6)
Eu—O9	2.486 (3)	C10—C11	1.373 (6)
Eu—O8	2.496 (3)	C10—H10A	0.9300
Eu—O5	2.540 (3)	C11—C12	1.374 (7)
Eu—O12	2.567 (3)	C11—H11A	0.9300
Eu—O2	2.743 (3)	C12—C13	1.384 (7)
Eu—O4	2.748 (3)	C12—C15	1.498 (7)
Eu—N3	2.906 (5)	C13—C14	1.369 (7)
Eu—N4	2.914 (4)	C13—H13A	0.9300
N1—C7	1.297 (5)	C14—H14A	0.9300
N1—C9	1.416 (5)	C15—H15A	0.9600
N1—H1A	0.8600	C15—H15B	0.9600
N2—C22	1.300 (5)	C15—H15C	0.9600
N2—C24	1.412 (5)	C16—C22	1.408 (6)
N2—H2A	0.8600	C16—C21	1.411 (6)
N3—O7	1.210 (5)	C16—C17	1.413 (5)
N3—O6	1.262 (6)	C17—C18	1.413 (6)
N3—O5	1.275 (6)	C18—C19	1.356 (6)
N4—O10	1.197 (5)	C19—C20	1.401 (7)
N4—O8	1.271 (5)	C19—H19A	0.9300
N4—O9	1.273 (5)	C20—C21	1.351 (6)
N5—O13	1.232 (5)	C20—H20A	0.9300
N5—O12	1.245 (5)	C21—H21A	0.9300
N5—O11	1.273 (5)	C22—H22A	0.9300
O1—C2	1.310 (5)	C23—H23A	0.9600
O2—C3	1.390 (5)	C23—H23B	0.9600
O2—C8	1.436 (5)	C23—H23C	0.9600
O3—C17	1.308 (5)	C24—C29	1.364 (6)
O4—C18	1.385 (5)	C24—C25	1.392 (6)
O4—C23	1.443 (6)	C25—C26	1.376 (7)
C1—C7	1.397 (6)	C25—H25A	0.9300
C1—C6	1.407 (6)	C26—C27	1.386 (7)
C1—C2	1.427 (5)	C26—H26A	0.9300
C2—C3	1.405 (6)	C27—C28	1.378 (7)
C3—C4	1.365 (6)	C27—C30	1.516 (7)
C4—C5	1.403 (7)	C28—C29	1.372 (7)
C4—H4A	0.9300	C28—H28A	0.9300
C5—C6	1.353 (7)	C29—H29A	0.9300
C5—H5A	0.9300	C30—H30A	0.9600
C6—H6A	0.9300	C30—H30B	0.9600
C7—H7A	0.9300	C30—H30C	0.9600
C8—H8A	0.9600		
			
O1—Eu—O3	156.86 (11)	N5—O12—Eu	93.7 (3)
O1—Eu—O6	126.05 (10)	C7—C1—C6	119.5 (4)
O3—Eu—O6	74.56 (11)	C7—C1—C2	121.2 (4)
O1—Eu—O11	77.22 (11)	C6—C1—C2	119.3 (4)
O3—Eu—O11	81.19 (11)	O1—C2—C3	121.2 (4)
O6—Eu—O11	128.28 (12)	O1—C2—C1	121.7 (4)
O1—Eu—O9	67.89 (10)	C3—C2—C1	117.1 (4)
O3—Eu—O9	114.59 (10)	C4—C3—O2	125.1 (4)
O6—Eu—O9	117.74 (12)	C4—C3—C2	122.5 (4)
O11—Eu—O9	113.83 (11)	O2—C3—C2	112.4 (4)
O1—Eu—O8	117.54 (10)	C3—C4—C5	119.5 (4)
O3—Eu—O8	73.74 (10)	C3—C4—H4A	120.2
O6—Eu—O8	78.23 (11)	C5—C4—H4A	120.2
O11—Eu—O8	136.58 (11)	C6—C5—C4	120.2 (5)
O9—Eu—O8	51.36 (10)	C6—C5—H5A	119.9
O1—Eu—O5	83.97 (12)	C4—C5—H5A	119.9
O3—Eu—O5	119.17 (12)	C5—C6—C1	121.3 (4)
O6—Eu—O5	51.38 (12)	C5—C6—H6A	119.3
O11—Eu—O5	152.48 (12)	C1—C6—H6A	119.3
O9—Eu—O5	76.25 (12)	N1—C7—C1	124.8 (4)
O8—Eu—O5	70.27 (11)	N1—C7—H7A	117.6
O1—Eu—O12	101.56 (11)	C1—C7—H7A	117.6
O3—Eu—O12	70.19 (10)	O2—C8—H8A	109.5
O6—Eu—O12	78.08 (12)	O2—C8—H8B	109.5
O11—Eu—O12	50.66 (11)	H8A—C8—H8B	109.5
O9—Eu—O12	164.00 (11)	O2—C8—H8C	109.5
O8—Eu—O12	140.88 (11)	H8A—C8—H8C	109.5
O5—Eu—O12	115.65 (12)	H8B—C8—H8C	109.5
O1—Eu—O2	61.53 (9)	C14—C9—C10	118.7 (4)
O3—Eu—O2	126.07 (9)	C14—C9—N1	118.9 (4)
O6—Eu—O2	72.06 (10)	C10—C9—N1	122.3 (4)
O11—Eu—O2	87.61 (11)	C11—C10—C9	120.2 (5)
O9—Eu—O2	118.15 (10)	C11—C10—H10A	119.9
O8—Eu—O2	135.78 (10)	C9—C10—H10A	119.9
O5—Eu—O2	65.64 (11)	C10—C11—C12	122.1 (5)
O12—Eu—O2	62.36 (10)	C10—C11—H11A	119.0
O1—Eu—O4	103.23 (9)	C12—C11—H11A	119.0
O3—Eu—O4	61.07 (9)	C11—C12—C13	116.8 (5)
O6—Eu—O4	128.88 (10)	C11—C12—C15	120.8 (5)
O11—Eu—O4	70.39 (11)	C13—C12—C15	122.4 (5)
O9—Eu—O4	65.81 (10)	C14—C13—C12	122.3 (5)
O8—Eu—O4	66.53 (10)	C14—C13—H13A	118.8
O5—Eu—O4	134.37 (11)	C12—C13—H13A	118.8
O12—Eu—O4	106.99 (10)	C13—C14—C9	119.9 (5)
O2—Eu—O4	156.14 (10)	C13—C14—H14A	120.0
O1—Eu—N3	106.12 (13)	C9—C14—H14A	120.0
O3—Eu—N3	96.52 (13)	C12—C15—H15A	109.5
O6—Eu—N3	25.40 (13)	C12—C15—H15B	109.5
O11—Eu—N3	147.00 (13)	H15A—C15—H15B	109.5
O9—Eu—N3	97.17 (13)	C12—C15—H15C	109.5
O8—Eu—N3	71.99 (11)	H15A—C15—H15C	109.5
O5—Eu—N3	25.98 (12)	H15B—C15—H15C	109.5
O12—Eu—N3	97.38 (13)	C22—C16—C21	119.9 (4)
O2—Eu—N3	67.05 (10)	C22—C16—C17	120.6 (4)
O4—Eu—N3	136.81 (10)	C21—C16—C17	119.5 (4)
O1—Eu—N4	93.14 (11)	O3—C17—C16	121.9 (4)
O3—Eu—N4	93.38 (11)	O3—C17—C18	120.3 (4)
O6—Eu—N4	99.32 (12)	C16—C17—C18	117.7 (4)
O11—Eu—N4	127.44 (11)	C19—C18—O4	126.4 (4)
O9—Eu—N4	25.74 (10)	C19—C18—C17	121.3 (4)
O8—Eu—N4	25.71 (10)	O4—C18—C17	112.3 (4)
O5—Eu—N4	72.97 (11)	C18—C19—C20	120.6 (4)
O12—Eu—N4	163.52 (11)	C18—C19—H19A	119.7
O2—Eu—N4	132.72 (10)	C20—C19—H19A	119.7
O4—Eu—N4	61.78 (10)	C21—C20—C19	119.8 (4)
N3—Eu—N4	85.49 (12)	C21—C20—H20A	120.1
C7—N1—C9	128.0 (4)	C19—C20—H20A	120.1
C7—N1—H1A	116.0	C20—C21—C16	121.1 (4)
C9—N1—H1A	116.0	C20—C21—H21A	119.5
C22—N2—C24	128.9 (4)	C16—C21—H21A	119.5
C22—N2—H2A	115.5	N2—C22—C16	122.7 (4)
C24—N2—H2A	115.5	N2—C22—H22A	118.7
O7—N3—O6	122.5 (6)	C16—C22—H22A	118.7
O7—N3—O5	121.1 (6)	O4—C23—H23A	109.5
O6—N3—O5	116.4 (4)	O4—C23—H23B	109.5
O7—N3—Eu	178.1 (5)	H23A—C23—H23B	109.5
O6—N3—Eu	55.6 (2)	O4—C23—H23C	109.5
O5—N3—Eu	60.8 (2)	H23A—C23—H23C	109.5
O10—N4—O8	122.2 (4)	H23B—C23—H23C	109.5
O10—N4—O9	121.7 (4)	C29—C24—C25	120.2 (4)
O8—N4—O9	116.2 (4)	C29—C24—N2	117.9 (4)
O10—N4—Eu	173.5 (3)	C25—C24—N2	121.9 (4)
O8—N4—Eu	58.5 (2)	C26—C25—C24	118.9 (5)
O9—N4—Eu	58.0 (2)	C26—C25—H25A	120.5
O13—N5—O12	122.7 (5)	C24—C25—H25A	120.5
O13—N5—O11	119.7 (5)	C25—C26—C27	121.7 (5)
O12—N5—O11	117.6 (4)	C25—C26—H26A	119.1
O13—N5—Eu	175.6 (4)	C27—C26—H26A	119.1
O12—N5—Eu	61.1 (2)	C28—C27—C26	117.5 (5)
O11—N5—Eu	56.5 (2)	C28—C27—C30	121.3 (5)
C2—O1—Eu	128.8 (3)	C26—C27—C30	121.2 (5)
C3—O2—C8	117.2 (3)	C29—C28—C27	122.0 (5)
C3—O2—Eu	114.8 (2)	C29—C28—H28A	119.0
C8—O2—Eu	127.1 (3)	C27—C28—H28A	119.0
C17—O3—Eu	128.5 (2)	C24—C29—C28	119.8 (5)
C18—O4—C23	116.4 (4)	C24—C29—H29A	120.1
C18—O4—Eu	114.2 (2)	C28—C29—H29A	120.1
C23—O4—Eu	128.9 (3)	C27—C30—H30A	109.5
N3—O5—Eu	93.3 (3)	C27—C30—H30B	109.5
N3—O6—Eu	99.0 (3)	H30A—C30—H30B	109.5
N4—O8—Eu	95.8 (2)	C27—C30—H30C	109.5
N4—O9—Eu	96.3 (2)	H30A—C30—H30C	109.5
N5—O11—Eu	97.9 (3)	H30B—C30—H30C	109.5
			
O1—Eu—N3—O6	145.0 (3)	O12—Eu—O5—N3	48.7 (3)
O3—Eu—N3—O6	−30.1 (3)	O2—Eu—O5—N3	87.2 (3)
O11—Eu—N3—O6	53.7 (4)	O4—Eu—O5—N3	−108.7 (3)
O9—Eu—N3—O6	−146.0 (3)	N4—Eu—O5—N3	−116.2 (3)
O8—Eu—N3—O6	−100.6 (3)	O7—N3—O6—Eu	−179.6 (4)
O5—Eu—N3—O6	177.6 (4)	O5—N3—O6—Eu	2.3 (4)
O12—Eu—N3—O6	40.7 (3)	O1—Eu—O6—N3	−42.9 (3)
O2—Eu—N3—O6	96.4 (3)	O3—Eu—O6—N3	148.9 (3)
O4—Eu—N3—O6	−84.0 (3)	O11—Eu—O6—N3	−146.0 (3)
N4—Eu—N3—O6	−123.0 (3)	O9—Eu—O6—N3	38.8 (3)
O1—Eu—N3—O5	−32.6 (3)	O8—Eu—O6—N3	72.7 (3)
O3—Eu—N3—O5	152.3 (3)	O5—Eu—O6—N3	−1.3 (2)
O6—Eu—N3—O5	−177.6 (4)	O12—Eu—O6—N3	−138.6 (3)
O11—Eu—N3—O5	−123.8 (3)	O2—Eu—O6—N3	−74.1 (3)
O9—Eu—N3—O5	36.4 (3)	O4—Eu—O6—N3	119.0 (3)
O8—Eu—N3—O5	81.8 (3)	N4—Eu—O6—N3	57.9 (3)
O12—Eu—N3—O5	−136.9 (3)	O10—N4—O8—Eu	−172.4 (4)
O2—Eu—N3—O5	−81.2 (3)	O9—N4—O8—Eu	6.2 (4)
O4—Eu—N3—O5	98.4 (3)	O1—Eu—O8—N4	−19.8 (3)
N4—Eu—N3—O5	59.4 (3)	O3—Eu—O8—N4	138.3 (3)
O1—Eu—N4—O8	162.5 (2)	O6—Eu—O8—N4	−144.5 (3)
O3—Eu—N4—O8	−39.7 (2)	O11—Eu—O8—N4	81.0 (3)
O6—Eu—N4—O8	35.2 (3)	O9—Eu—O8—N4	−3.7 (2)
O11—Eu—N4—O8	−121.2 (2)	O5—Eu—O8—N4	−91.6 (3)
O9—Eu—N4—O8	173.4 (4)	O12—Eu—O8—N4	161.7 (2)
O5—Eu—N4—O8	79.8 (2)	O2—Eu—O8—N4	−96.2 (3)
O12—Eu—N4—O8	−44.3 (5)	O4—Eu—O8—N4	73.4 (2)
O2—Eu—N4—O8	109.3 (3)	N3—Eu—O8—N4	−119.0 (3)
O4—Eu—N4—O8	−94.2 (3)	O10—N4—O9—Eu	172.4 (4)
N3—Eu—N4—O8	56.5 (3)	O8—N4—O9—Eu	−6.3 (4)
O1—Eu—N4—O9	−10.9 (2)	O1—Eu—O9—N4	168.2 (3)
O3—Eu—N4—O9	146.9 (2)	O3—Eu—O9—N4	−36.9 (3)
O6—Eu—N4—O9	−138.2 (2)	O6—Eu—O9—N4	48.0 (3)
O11—Eu—N4—O9	65.4 (3)	O11—Eu—O9—N4	−127.9 (2)
O8—Eu—N4—O9	−173.4 (4)	O8—Eu—O9—N4	3.7 (2)
O5—Eu—N4—O9	−93.6 (3)	O5—Eu—O9—N4	79.2 (2)
O12—Eu—N4—O9	142.3 (4)	O12—Eu—O9—N4	−141.0 (4)
O2—Eu—N4—O9	−64.1 (3)	O2—Eu—O9—N4	131.5 (2)
O4—Eu—N4—O9	92.4 (2)	O4—Eu—O9—N4	−74.8 (2)
N3—Eu—N4—O9	−116.9 (3)	N3—Eu—O9—N4	63.7 (3)
O1—Eu—N5—O12	−121.2 (3)	O13—N5—O11—Eu	177.2 (3)
O3—Eu—N5—O12	75.4 (2)	O12—N5—O11—Eu	−3.4 (4)
O6—Eu—N5—O12	5.6 (3)	O1—Eu—O11—N5	−115.0 (3)
O11—Eu—N5—O12	176.6 (4)	O3—Eu—O11—N5	73.4 (2)
O9—Eu—N5—O12	−174.3 (2)	O6—Eu—O11—N5	11.2 (3)
O8—Eu—N5—O12	94.1 (3)	O9—Eu—O11—N5	−173.5 (2)
O5—Eu—N5—O12	−41.3 (3)	O8—Eu—O11—N5	128.3 (2)
O2—Eu—N5—O12	−60.7 (2)	O5—Eu—O11—N5	−66.9 (4)
O4—Eu—N5—O12	135.5 (2)	O12—Eu—O11—N5	1.9 (2)
N3—Eu—N5—O12	−13.0 (3)	O2—Eu—O11—N5	−53.7 (2)
N4—Eu—N5—O12	144.6 (3)	O4—Eu—O11—N5	135.7 (3)
O1—Eu—N5—O11	62.2 (3)	N3—Eu—O11—N5	−15.0 (4)
O3—Eu—N5—O11	−101.2 (3)	N4—Eu—O11—N5	160.9 (2)
O6—Eu—N5—O11	−171.0 (2)	O13—N5—O12—Eu	−177.4 (4)
O9—Eu—N5—O11	9.1 (3)	O11—N5—O12—Eu	3.2 (4)
O8—Eu—N5—O11	−82.5 (3)	O1—Eu—O12—N5	60.7 (3)
O5—Eu—N5—O11	142.1 (3)	O3—Eu—O12—N5	−96.8 (3)
O12—Eu—N5—O11	−176.6 (4)	O6—Eu—O12—N5	−174.4 (3)
O2—Eu—N5—O11	122.7 (3)	O11—Eu—O12—N5	−1.9 (2)
O4—Eu—N5—O11	−41.1 (3)	O9—Eu—O12—N5	13.6 (5)
N3—Eu—N5—O11	170.4 (2)	O8—Eu—O12—N5	−120.6 (3)
N4—Eu—N5—O11	−32.0 (4)	O5—Eu—O12—N5	149.6 (2)
O3—Eu—O1—C2	125.4 (3)	O2—Eu—O12—N5	109.7 (3)
O6—Eu—O1—C2	−24.5 (4)	O4—Eu—O12—N5	−47.1 (3)
O11—Eu—O1—C2	103.9 (3)	N3—Eu—O12—N5	168.9 (3)
O9—Eu—O1—C2	−133.5 (4)	N4—Eu—O12—N5	−91.9 (5)
O8—Eu—O1—C2	−119.9 (3)	Eu—O1—C2—C3	−9.3 (6)
O5—Eu—O1—C2	−55.9 (3)	Eu—O1—C2—C1	169.7 (3)
O12—Eu—O1—C2	59.1 (3)	C7—C1—C2—O1	−2.1 (6)
O2—Eu—O1—C2	9.6 (3)	C6—C1—C2—O1	179.4 (4)
O4—Eu—O1—C2	169.9 (3)	C7—C1—C2—C3	177.0 (4)
N3—Eu—O1—C2	−42.2 (4)	C6—C1—C2—C3	−1.5 (6)
N4—Eu—O1—C2	−128.4 (3)	C8—O2—C3—C4	−3.0 (6)
O1—Eu—O2—C3	−9.0 (3)	Eu—O2—C3—C4	−173.0 (4)
O3—Eu—O2—C3	−163.0 (2)	C8—O2—C3—C2	178.4 (4)
O6—Eu—O2—C3	142.6 (3)	Eu—O2—C3—C2	8.4 (4)
O11—Eu—O2—C3	−85.8 (3)	O1—C2—C3—C4	−179.9 (4)
O9—Eu—O2—C3	30.1 (3)	C1—C2—C3—C4	1.1 (6)
O8—Eu—O2—C3	92.4 (3)	O1—C2—C3—O2	−1.2 (6)
O5—Eu—O2—C3	87.6 (3)	C1—C2—C3—O2	179.7 (4)
O12—Eu—O2—C3	−131.8 (3)	O2—C3—C4—C5	−179.1 (4)
O4—Eu—O2—C3	−63.4 (4)	C2—C3—C4—C5	−0.7 (7)
N3—Eu—O2—C3	115.9 (3)	C3—C4—C5—C6	0.7 (8)
N4—Eu—O2—C3	56.4 (3)	C4—C5—C6—C1	−1.3 (8)
O1—Eu—O2—C8	−177.9 (4)	C7—C1—C6—C5	−176.8 (5)
O3—Eu—O2—C8	28.1 (4)	C2—C1—C6—C5	1.7 (7)
O6—Eu—O2—C8	−26.3 (3)	C9—N1—C7—C1	−178.1 (4)
O11—Eu—O2—C8	105.4 (4)	C6—C1—C7—N1	177.3 (4)
O9—Eu—O2—C8	−138.8 (3)	C2—C1—C7—N1	−1.2 (7)
O8—Eu—O2—C8	−76.5 (4)	C7—N1—C9—C14	170.5 (5)
O5—Eu—O2—C8	−81.3 (4)	C7—N1—C9—C10	−9.7 (7)
O12—Eu—O2—C8	59.3 (3)	C14—C9—C10—C11	−1.2 (7)
O4—Eu—O2—C8	127.7 (3)	N1—C9—C10—C11	178.9 (4)
N3—Eu—O2—C8	−52.9 (4)	C9—C10—C11—C12	1.2 (8)
N4—Eu—O2—C8	−112.5 (3)	C10—C11—C12—C13	−0.7 (8)
O1—Eu—O3—C17	67.9 (4)	C10—C11—C12—C15	−178.4 (5)
O6—Eu—O3—C17	−137.0 (3)	C11—C12—C13—C14	0.1 (8)
O11—Eu—O3—C17	89.1 (3)	C15—C12—C13—C14	177.8 (5)
O9—Eu—O3—C17	−23.1 (4)	C12—C13—C14—C9	−0.1 (8)
O8—Eu—O3—C17	−55.0 (3)	C10—C9—C14—C13	0.7 (7)
O5—Eu—O3—C17	−110.6 (3)	N1—C9—C14—C13	−179.5 (5)
O12—Eu—O3—C17	140.4 (3)	Eu—O3—C17—C16	164.4 (3)
O2—Eu—O3—C17	169.6 (3)	Eu—O3—C17—C18	−16.8 (5)
O4—Eu—O3—C17	16.7 (3)	C22—C16—C17—O3	2.9 (6)
N3—Eu—O3—C17	−124.1 (3)	C21—C16—C17—O3	−179.5 (4)
N4—Eu—O3—C17	−38.2 (3)	C22—C16—C17—C18	−175.9 (4)
O1—Eu—O4—C18	−176.7 (3)	C21—C16—C17—C18	1.7 (6)
O3—Eu—O4—C18	−15.1 (2)	C23—O4—C18—C19	7.1 (6)
O6—Eu—O4—C18	18.2 (3)	Eu—O4—C18—C19	−166.2 (4)
O11—Eu—O4—C18	−105.7 (3)	C23—O4—C18—C17	−173.1 (4)
O9—Eu—O4—C18	125.2 (3)	Eu—O4—C18—C17	13.6 (4)
O8—Eu—O4—C18	68.7 (3)	O3—C17—C18—C19	178.7 (4)
O5—Eu—O4—C18	88.7 (3)	C16—C17—C18—C19	−2.4 (6)
O12—Eu—O4—C18	−70.1 (3)	O3—C17—C18—O4	−1.1 (5)
O2—Eu—O4—C18	−129.5 (3)	C16—C17—C18—O4	177.8 (4)
N3—Eu—O4—C18	51.4 (3)	O4—C18—C19—C20	−178.7 (4)
N4—Eu—O4—C18	96.8 (3)	C17—C18—C19—C20	1.5 (7)
O1—Eu—O4—C23	10.9 (4)	C18—C19—C20—C21	0.2 (7)
O3—Eu—O4—C23	172.6 (4)	C19—C20—C21—C16	−0.9 (7)
O6—Eu—O4—C23	−154.1 (4)	C22—C16—C21—C20	177.5 (4)
O11—Eu—O4—C23	82.0 (4)	C17—C16—C21—C20	−0.1 (7)
O9—Eu—O4—C23	−47.1 (4)	C24—N2—C22—C16	177.3 (4)
O8—Eu—O4—C23	−103.6 (4)	C21—C16—C22—N2	−178.2 (4)
O5—Eu—O4—C23	−83.6 (4)	C17—C16—C22—N2	−0.6 (6)
O12—Eu—O4—C23	117.6 (4)	C22—N2—C24—C29	−169.5 (5)
O2—Eu—O4—C23	58.2 (5)	C22—N2—C24—C25	10.2 (7)
N3—Eu—O4—C23	−120.9 (4)	C29—C24—C25—C26	1.0 (7)
N4—Eu—O4—C23	−75.5 (4)	N2—C24—C25—C26	−178.7 (4)
O7—N3—O5—Eu	179.7 (4)	C24—C25—C26—C27	−0.1 (7)
O6—N3—O5—Eu	−2.2 (4)	C25—C26—C27—C28	−0.8 (8)
O1—Eu—O5—N3	148.7 (3)	C25—C26—C27—C30	178.3 (5)
O3—Eu—O5—N3	−31.9 (3)	C26—C27—C28—C29	0.9 (8)
O6—Eu—O5—N3	1.3 (2)	C30—C27—C28—C29	−178.2 (5)
O11—Eu—O5—N3	101.8 (3)	C25—C24—C29—C28	−0.9 (7)
O9—Eu—O5—N3	−142.7 (3)	N2—C24—C29—C28	178.8 (5)
O8—Eu—O5—N3	−89.2 (3)	C27—C28—C29—C24	0.0 (8)

### Hydrogen-bond geometry (Å, °)

**Table:**

*D*—H···*A*	*D*—H	H···*A*	*D*···*A*	*D*—H···*A*
N1—H1A···O1	0.86	1.95	2.634 (4)	136
N2—H2A···O3	0.86	1.86	2.569 (4)	139
